# Is There a Minimum Effective Dose for Vascular Occlusion During Blood Flow Restriction Training?

**DOI:** 10.3389/fphys.2022.838115

**Published:** 2022-04-08

**Authors:** Arpan Das, Bruce Paton

**Affiliations:** Institute of Sports, Exercise and Health, Department of Medical Sciences, University College of London, London, United Kingdom

**Keywords:** BFR, strength training, bloodflow restriction training, rehabilitation, kaatsu, 1RM, one repetition maximum, dosage

## Abstract

**Background:**

Blood flow restriction (BFR) training at lower exercise intensities has a range of applications, allowing subjects to achieve strength and hypertrophy gains matching those training at high intensity. However, there is no clear consensus on the percentage of limb occlusion pressure [%LOP, expressed as a % of the pressure required to occlude systolic blood pressure (SBP)] and percentage of one repetition max weight (%1RM) required to achieve these results. This review aims to explore what the optimal and minimal combination of LOP and 1RM is for significant results using BFR.

**Method:**

A literature search using PubMed, Scopus, Wiley Online, Springer Link, and relevant citations from review papers was performed, and articles assessed for suitability. Original studies using BFR with a resistance training exercise intervention, who chose a set %LOP and %1RM and compared to a non-BFR control were included in this review.

**Result:**

Twenty-one studies met the inclusion criteria. %LOP ranged from 40 to 150%. %1RM used ranged from 15 to 80%. Training at 1RM ≤20%, or ≥ 80% did not produce significant strength results compared to controls. Applying %LOP of ≤50% and ≥ 80% did not produce significant strength improvement compared to controls. This may be due to a mechanism mediated by lactate accumulation, which is facilitated by increased training volume and a moderate exercise intensity.

**Conclusion:**

Training at a minimum of 30 %1RM with BFR is required for strength gains matching non-BFR high intensity training. Moderate intensity training (40–60%1RM) with BFR may produce results exceeding non-BFR high intensity however the literature is sparse. A %LOP of 50–80% is optimal for BFR training.

## Introduction

Blood flow restriction (BFR) training is a novel area of research within the strength and conditioning world. A pressurized cuff is applied proximally to the muscle trained and is inflated to partially occlude arterial blood flow but fully occlude venous return. This training method shows similar results to standard high intensity resistance training in both hypertrophy and strength at much lower exercise intensities. An increase in maximal voluntary contraction of 26% was reported in subjects training at 40% of one repetition max (1RM) with a 250 mmHg cuff applied to their thigh after 4 weeks, versus a control group undergoing the same intervention without limb occlusion who saw no significant change (Shinohara et al., 1998). An increase in thigh muscle cross sectional area (CSA) of 10.3% was found in subjects training with BFR at total limb occlusion pressure (LOP) at 10–20% 1RM for 8 weeks, whereas the control group on the same program without BFR had no significant hypertrophy (Takarada et al., 2004). This training method has broad applications - not only to strength athletes and bodybuilders but also to those who may find high intensity training difficult, such as people with osteoporosis, the elderly, or those going through exercise rehabilitation. These groups may benefit from heavy load training (Watson et al., 2015) but struggle to train at high intensities due to fracture risk, pain (Csapo and Alegre, 2016), or due to healing tissue which may be vulnerable to loading. Adverse side effects such as acute hypotension and reduction in vascular compliance have been observed with BFR training (Loenneke et al., 2013), however previously anticipated complications such as deep vein thrombosis and rhabdomyolysis (Vanwye et al., 2017; Minniti et al., 2020) are extremely rare indicating that BFR appears to be safe.

Exercise discomfort, post training soreness and reduced exercise volume appear to be associated with higher cuff pressures (Brandner and Warmington, 2017). Therefore, it would be advantageous to find a minimum effective pressure of vascular occlusion which would still grant significant strength adaptations that meet or even exceed those achievable with standard high intensity resistance training. While many papers have studied the effects of BFR at set applied pressures, less have explored the effects of BFR at a percentage of individual LOP. Individualizing the applied pressure based on LOP should theoretically produce a more effective and uniform physiological stress than a standardized set pressure. However, a recent review found that over 86% of studies of BFR training included no justification of the LOP chosen in their methodology (Clarkson et al., 2020).

This systematic review aims to determine a minimal and optimal applied vascular occlusion pressure during BFR training. It will also seek to determine a minimum exercise intensity (%1RM), volume load and total repetitions required to produce significant strength improvements. This will guide blood flow restricted exercise prescription for recreational strength training and exercise rehabilitation. Determining minimum effective pressure will allow us to avoid adverse effects and minimize exercise discomfort and delayed onset soreness.

## Method

This systematic review was conducted using the Preferred Reporting Items for Systematic Reviews and Meta-Analyzes (PRISMA) guidelines (Moher et al., 2009).

### Search Method

**FIGURE 1:**
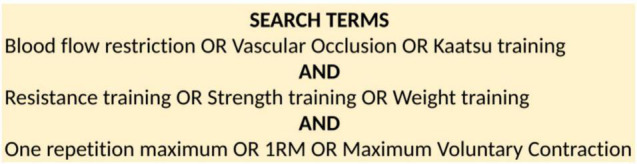
Search terms with alternatives used for literature search.

Online research databases PubMed, Scopus, Wiley Online Library and Springer Link were searched from May 2021. Search terms included “BFR training,” “resistance training,” and “1RM” as well as variations of these terms (see Figure 1). Relevant citations from reference lists of BFR reviews were also included. Where possible, filters were applied including “available in English,” “research article,” and “full text available.” No set range of publication date was used.

### Inclusion Criteria

Search results were saved into Zotero and duplicates removed. Single screening was performed by title, abstract and full text. Inclusion criteria were;

(1) Text available in English.
(2) Full text was available.
(3) Original research papers only. Systematic and narrative reviews, editorials, abstracts, and discussions of previous original work were excluded. Relevant citations from reference lists of these papers were included.
(4) BFR must have been included as part of an ongoing exercise intervention. Studies that investigated the acute effect of BFR were excluded. The intervention must have lasted at least 4 weeks.
(5) Exercise interventions were resistance training based. Aerobic, cardiovascular, and walking studies were excluded as we were investigating the effect of BFR on strength outcomes from resistance training.
(6) BFR method used had to be continuous - i.e., the BFR cuff remained on the participant throughout the exercise session with no breaks (as opposed to intermittent, where the participant removes the cuff during breaks).
(7) A non-BFR using control group was required who were also involved in an exercise intervention. Studies with non-exercising controls were excluded, as well as studies that only compared different %LOP without a non-BFR control.
(8) Initially, only studies using a control that were training using a standard “high intensity” strength training protocol as per the American College of Sports Medicine guidelines (Liguori, 2021) were included (70–80% 1RM, 3–4 sets of 8–12 repetitions). Studies that used a control group exercising at the same exercise intensity and volume as the BFR test group were included separately. As BFR studies tend to use lower exercise intensities (20–30%1RM), this would allow comparison of BFR as an independent variable against non-BFR groups training with the same protocol.
(9) Only papers that used a set percentage of individual LOP were included. Appropriate measurement of LOP should have therefore been demonstrated. Papers that used a standardized pressure of mmHg for all participants were excluded as actual dosage of BFR could vary considerably between participants.
(10) Chosen percentage of LOP and %1RM stayed constant throughout the intervention period.
(11) 1RM strength change was included as an outcome measure with pre and post values to calculate % change and effect size.
(12) Methodology included full description of exercise intervention for both the BFR testing and control groups.
(13) Participant group had no prior relevant medical history which could affect result i.e., people with cardiovascular/orthopedic/rheumatological conditions or undergoing exercise rehabilitation, in order to prevent their symptoms and range of severity from confounding the results from these studies.
(14) Participants could be of any age.
(15) Participants could be of any gender.
(16) Participants could have any duration prior training experience.

We did not control for age or gender, but recognize that these factors may affect response to bloodflow restriction due to vascular differences (see section “Limitations”). Ideally, we would have liked to analyze sub groups in populations based on gender, age, and training experience however the sparseness of the literature at this time would not have allowed for adequate total sample size to make meaningful conclusions.

**FIGURE 2:**
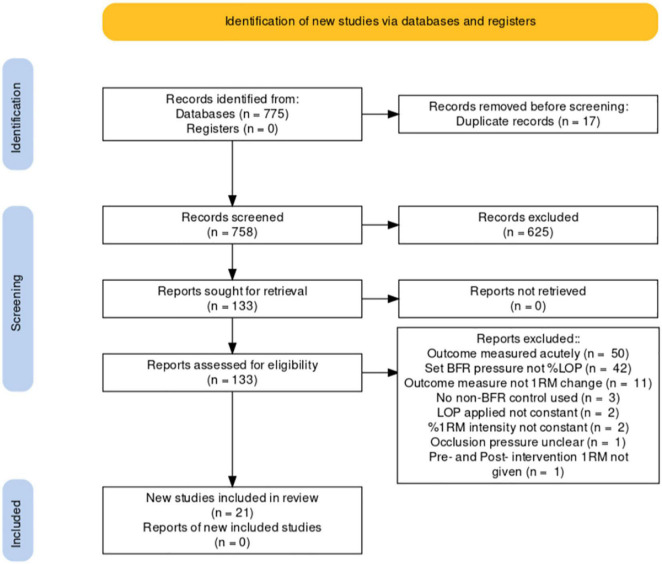
PRISMA flowchart of exclusion process.

Exclusions are identified in the PRISMA flowchart below (see Figure 2).

### Study Quality

Study quality and study bias were evaluated. Quality and bias assessment was performed using an original tool created for BFR studies (Clarkson et al., 2020; see Appendix Figure 3). Criteria in this tool included experimental design reporting, participant choice and selection, as well as clarity and reproducibility of method. The maximum score in this tool is thirteen, with papers scoring below seven deemed to be of lesser quality or high bias risk. Lower score did not result in exclusion but enabled measurement of the overall quality of BFR literature. Level of evidence was evaluated using the Oxford Centre for Evidence Based Medicine tool (Centre for Evidence-Based Medicine [CEBM], 2009) which assesses studies on the quality of randomization, blinding, equality of intervention, and handling of participant dropout.

### Data Extraction and Analysis

On completion of the initial screening, data from the original research were extracted including participant demographic, sample size, muscle group tested, type and length of exercise intervention (BFR and control groups), % of LOP applied (%LOP), and exercise intensity (%1RM). %1RM change from baseline to completion for BFR and control group was calculated from available data if %/Δ change was not included in study results. Effect size was calculated using Cohen’s d (see Appendix Figures 4, 5).

## Results

### Search Results

A total of 775 articles were identified in databases and reference lists up to May 2021 (Scopus *n* = 84, PubMed *n* = 42, Wiley Online Library *n* = 187, Science Direct *n* = 157, Springer Link *n* = 253, Reference lists *n* = 52). Seventeen duplicates were removed. Of the 758 remaining papers, 625 were single-screened and excluded from title or abstract. One hundred thirty-three papers were then evaluated using a *pro forma* based on my inclusion criteria. From that *pro forma* 112 were excluded leaving 21 (Figure 2).

### Study Characteristics

Key characteristics of the included studies were sample size, %LOP, %1RM, exercise intervention design and length, and muscle group targeted (see Appendix Figure 4). Control group was categorized as a high intensity (70–80% 1RM) protocol or an intensity and volume matched protocol to the BFR group. The 21 studies included had a total sample size of 590. In the 21 studies chosen, 48 different protocols were tested with different combinations of %LOP, %1RM and target muscle group. Table 1 shows the distribution of %LOP across the 48 protocols. Table 2 shows the distribution of %1RM intensity chosen for the BFR groups. Table 3 shows the distribution of muscle groups tested.

**TABLE 1:** Distribution of applied LOP across the 48 protocols.

%LOP applied	Number of protocols
≥100 (Max 150)	9 (Laurentino et al., 2008; Manimmanakorn et al., 2013; Cook et al., 2017; Biazon et al., 2019; de Lemos Muller et al., 2019)
80	10 (Laurentino et al., 2012; Lixandrão et al., 2015; Jessee et al., 2018; de Lemos Muller et al., 2019; Neto et al., 2019)
70	1 (Ruaro et al., 2019)
60	11 (May et al., 2018; Brandner et al., 2019; Gavanda et al., 2020; Mendonca et al., 2021)
50	9 (Fahs et al., 2015; Vechin et al., 2015; Kim et al., 2017; Bemben et al., 2019; Centner et al., 2019)
40–45	7 (Lixandrão et al., 2015; Jessee et al., 2018; Letieri et al., 2018; Hill et al., 2020)

**TABLE 2:** Distribution of %1RM intensity for BFR training across the 48 protocols.

%1RM intensity chosen	Number of protocols
80	3 (Laurentino et al., 2008; Lixandrão et al., 2015; Biazon et al., 2019)
50–60	3 (Laurentino et al., 2008; Cook et al., 2017; May et al., 2018)
40	2 (Cook et al., 2017; Ruaro et al., 2019)
30	20 (Fahs et al., 2015; Cook et al., 2017; Kim et al., 2017; Letieri et al., 2018; May et al., 2018; Bemben et al., 2019; de Lemos Muller et al., 2019; Gavanda et al., 2020; Hill et al., 2020)
15–20	20 (Laurentino et al., 2012; Manimmanakorn et al., 2013; Lixandrão et al., 2015; Jessee et al., 2018; Brandner et al., 2019; Neto et al., 2019; Clarkson et al., 2020; Mendonca et al., 2021)

**TABLE 3:** Distribution of exercise choice/muscle group tested across the 48 protocols.

Exercise choice	Number of protocols
Leg extension/quadricep extension	20 (Laurentino et al., 2008, 2012; Manimmanakorn et al., 2013; Fahs et al., 2015; Cook et al., 2017; Jessee et al., 2018; Letieri et al., 2018; May et al., 2018; Bemben et al., 2019; Biazon et al., 2019; Brandner et al., 2019; de Lemos Muller et al., 2019; Mendonca et al., 2021)
Elbow flexion/bicep curl	7 (Kim et al., 2017; May et al., 2018; Bemben et al., 2019; Brandner et al., 2019; de Lemos Muller et al., 2019; Neto et al., 2019; Hill et al., 2020)
Leg flexion/hamstring curl	5 (Cook et al., 2017; Letieri et al., 2018; May et al., 2018; Bemben et al., 2019)
Squat/leg press	4 (Vechin et al., 2015; Cook et al., 2017; Bemben et al., 2019; Brandner et al., 2019)
Bench press/chest press	3 (Bemben et al., 2019; Brandner et al., 2019; Neto et al., 2019)
Calf raise	3 (Brandner et al., 2019; Gavanda et al., 2020; Mendonca et al., 2021)
Lateral pulldown/front Pulldown	2 (Bemben et al., 2019; Neto et al., 2019)
Elbow extension/tricep extension	1 (Neto et al., 2019)
Seated row	1 (Brandner et al., 2019)
Wrist flexion/grip strength	1 (Ruaro et al., 2019)

Ten protocols used control groups who trained at the same exercise intensity and volume as the BFR test group (Manimmanakorn et al., 2013; Fahs et al., 2015; May et al., 2018; Neto et al., 2019; Hill et al., 2020). The most common exercise load dosage was four sets of 30,15,15,15 repetitions (19 protocols), with most citing Loenneke et al.’s (2012) paper as their reasoning (Loenneke et al., 2012).

Most studies used participants who were not currently undertaking resistance training with only 5 studies using resistance trained participants. Almost half the studies (10/21) used solely male participants, with a third of studies (7/21) using untrained young men. The remaining studies used mixed participants or female only samples (7 and 4, respectively). Almost three quarters (15/21) of the studies used young (age 20–30) participants, the remaining using participants over 50 years old (5/21) or middle aged (40–50 years old) participants (1/21). Papers including participants with existing injuries or medical conditions were intentionally excluded, however this could be studied in a further review.

### Study Findings

#### Limb Occlusion Pressure

**FIGURE 3:**
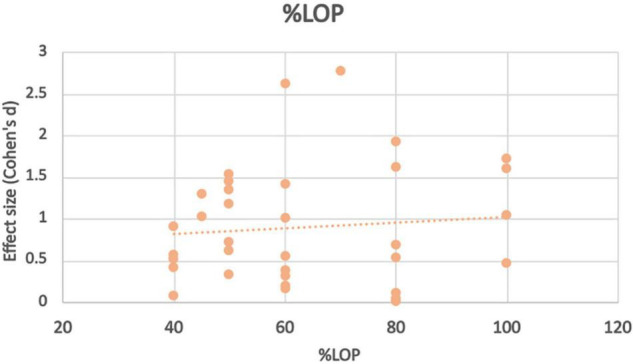
Effect size observed at different%LOP applied during BFR exercise intervention with trendline.

At pressures at or exceeding total LOP (100–150%), high intensity training non-BFR groups (70–80%1RM) showed significantly greater strength improvements at all exercise intensities. At moderate to high intensities (60–80%) there was no statistically significant difference in strength gain found between BFR and non-BFR. BFR subjects in this study (Laurentino et al., 2008)however trained using a standard moderate repetition protocol rather than the higher repetition lower intensity protocol. At 80% LOP, BFR subjects who trained at very low intensities (15–20% 1RM) had statistically significantly lower strength gains compared to the high intensity control. One study however found that 20% 1RM was enough to produce strength improvements higher (but not statistically higher) than a high intensity non-BFR control. 30% 1RM at this BFR pressure produced similar strength improvements between the two groups. The study that tested 70% LOP found that strength (grip) was significantly higher in those training at 40% 1RM than non-BFR controls. At 60% LOP, non-BFR training produced better results for all exercises at 20% 1RM. This difference in strength gain between the groups was larger in exercises where the major contributing muscle group was too proximal to be occluded (bench press, seated row, and barbell squat) and less where the target muscle could be occluded (leg extension, bicep curl and calf raise). At 30% LOP, strength improvements were higher in the BFR group however there was not a statistical difference. At 50% LOP and 30% 1RM, interestingly Bemben (Bemben et al., 2019) found greater strength improvements in the BFR group in exercises where the target muscle could be occluded, such as bicep curl, knee extension and knee flexion, but greater improvements in the non-BFR group in exercises where the primary muscle was too proximal such as lateral pulldown and bench press. At 20% however, the non-BFR group had significantly greater strength improvements. Finally at 40% LOP, Lixandrão et al. (2015), Jessee et al. (2018) and Letieri et al. (2018) found significantly lower strength improvements for the BFR group compared to non-BFR at all ranges of exercise intensities (80, 40, 30, 20, and 15%, respectively). Applied LOP does not appear to have the most significant effect on BFR strength adaptation, however extremes of pressures (<30%, >80%) appear to bring the worst results. Effect size varies wildly across amounts of applied LOP (see Figure 3), showing no true trend based on the studies chosen at this time.

#### Exercise Intensity

**FIGURE 4:**
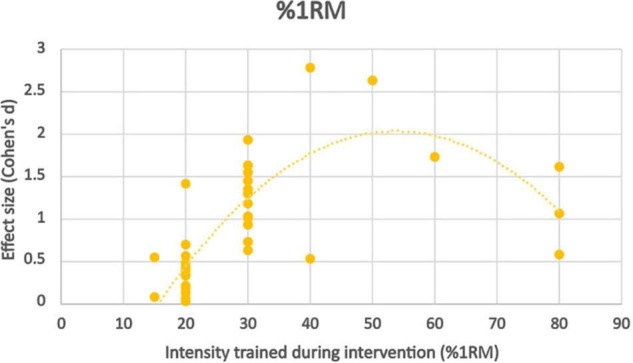
Effect size observed at different%1RM trained during BFR exercise intervention with trendline.

Training at 80% 1RM with low pressure BFR (40% LOP) produced significantly worse strength improvements than controls, although comparable results with high pressure BFR (100% LOP). Both studies looking at intensities of 50–60% 1RM with BFR found similar strength improvements to high intensity non-BFR but only at very high pressure (Laurentino et al., 2012; Cook et al., 2017) (100–150% LOP). Interestingly 40% 1RM with BFR produces significantly greater strength improvements when moderate pressure was applied (70% LOP), but significantly lower strength improvements at low-moderate pressure (40% LOP). 30% 1RM at BFR pressures exceeding total artery occlusion pressure produced significantly worse strength results than non-BFR controls. 30% 1RM at 80% LOP produced similar results between the BFR and non-BFR groups. At this exercise intensity and BFR pressure of 50–60% LOP, the BFR groups strength improvement exceeded the non-BFR group in exercises where the primary muscle group could be occluded. At 45% LOP, there was a significantly weaker effect than seen in the same study at 80% LOP. At very low intensities (15–20% 1RM), the non-BFR groups had significantly stronger strength improvements compared to the BFR groups at all pressures (100, 80, 60, 50, and 40%), the disparity seen most at the lower pressures applied (Figure 4).

In all studies where the control group trained at an exercise intensity and training volume matched to the low intensity BFR group, the BFR group had significantly greater strength improvements than the non-BFR group. In one study however, neither group made statistically significant strength improvements from baseline training at 20% 1RM.

Effect size appears to peak somewhere in the moderate intensity (50–60% 1RM) zone, however there is a significant skewness across distribution toward the lowest intensities due to very few studies using higher intensity protocols. Although there appears to be a trend, further work should be done in this area to verify whether this rings true.

**FIGURE 5:**
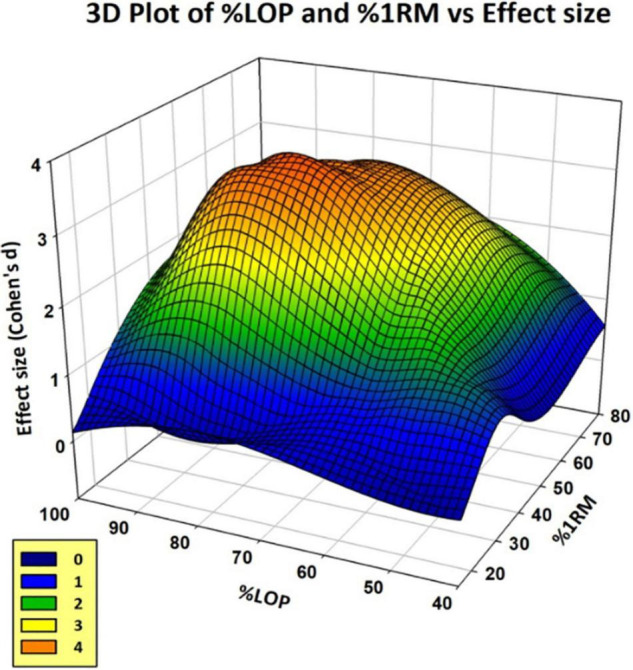
3D plot of %LOP (*x*-axis), %1RM (*y*-axis), and Effect Size (*z*-axis) for the 48 protocols.

Figure 5 shows the effect size plotted at each combination of %LOP and %1RM for the BFR test groups. Effect sizes were calculated for each combination of %LOP and %1RM from data presented in each study. Some studies were excluded as they did not present pre- and post-intervention, only presenting Δ strength improvement or percentage of strength increase, which prevented effect size from being calculated. As demonstrated, extremes of applied LOP and %1RM intensity respectively prove far less successful in generating significant improvements in strength gains using BFR training. However, combinations of moderate amounts of LOP and %1RM appear to show substantial effect. The “orange zone” of the graph shows the area of maximal effect. This appears to be between 60–80% LOP and 40–60% 1RM.

## Discussion

The purpose of this review was to determine the minimal and optimal amount of %LOP, exercise intensity and overall exercise dosage to elicit significant improvements in strength from BFR training. Results show that exercise intensity of 20% 1RM or below with BFR did not significantly increase strength compared to controls training at high intensity without BFR (70–80% 1RM). However, they did increase strength significantly against intensity matched controls. 30% 1RM with BFR appears to match high intensity non-BFR controls in strength gains, and 40%1RM appears to produce results that exceed high intensity controls.

### Metabolic Stress

The above findings from clinical exercise-based studies may be explained by mechanism based BFR studies, with metabolic pathways in muscle affected differently by different dosages of LOP and exercise load/intensity. Suga et al. (2010) tested intramuscular levels of pH, phosphocreatine (PCr) and dihydrogen phosphate (H2PO4) in subjects training at 20, 30, and 40% 1RM with moderate BFR (130 mmHg), and 20% with high BFR (200 mmHg) against controls training at low and high intensities (20 and 80%, respectively). They found that 30% 1RM with moderate BFR caused drops in pH, PCr, and H2PO4 that matched the high intensity control, whereas 40% 1RM exceeded the change in these metabolic markers. Conversely, there was no significant difference in metabolite change between the 20%1RM group with moderate BFR and high BFR. This may explain why Lixandrao’s study found that effect size favored 40% 1RM over 20%1RM at all LOPs, however there was no significant difference between 40 and 80% LOP at equal %1RM (Lixandrão et al., 2015). This reinforces the hypothesis that beyond a certain %LOP, there are no additional dose benefits to increased pressure and that exercise intensity becomes the primary variable for strength improvement.

The lower pH may be explained by increased levels of lactate during increasing exercise intensity. Goto et al. (2005) found that in two groups performing the same volume of exercise per session (3–5 sets of 10RM), the group who were given a 30 s break halfway through each set accumulated less lactate. They observed an increased growth hormone (GH) response and catecholamine release in the non-break group. The non-break group in this study consequently had a larger increase in lean mass, increased muscle CSA and increased leg extension at 12 weeks (Goto et al., 2005). Subjects training with BFR have been shown to have increased levels of GH, lactate, and noradrenaline post training than controls (Takarada et al., 2000). The increased lactate may be attributed to not only the impedance of venous return from the BFR cuff, but also reduced oxygen delivery due to partially occluded arterial flow, causing a hypoxic environment (Killinger et al., 2019). Lactate then accumulates in the muscle due to reduced aerobic respiration, at higher levels with increasing exercise intensity (Mazzeo et al., 1986). The increase in adrenaline seen in BFR training also contributes to lactate accumulation, as adrenaline reduces lactate uptake and metabolism in muscle *via* a β-adrenergic mechanism (Hamann et al., 2001).

### Lactic Acid, pH, and Growth Hormone Stimulation

Kraemer’s review found a strong association between muscle acidosis and GH release which he attributed to increased lactic acid (Kraemer and Ratamess, 2005). Increased GH was also associated with increased volume of exercise, as there was less time for lactate to be metabolised (Kraemer and Ratamess, 2005). In Reeves study, similar levels of lactate were observed between subjects training at 30%1RM with a BFR of 20 mmHg below SBP and controls training at 70%1RM without BFR. However, the GH response in the BFR group was fourfold of the non-BFR group (Reeves et al., 2006). The correlation between pH and GH was later confirmed by a study that found that subjects given an alkaline solution prior to a high intensity cycle trial had an attenuated GH response compared to controls given a neutral placebo (Gordon et al., 1994).

### Growth Hormone

**FIGURE 6:**
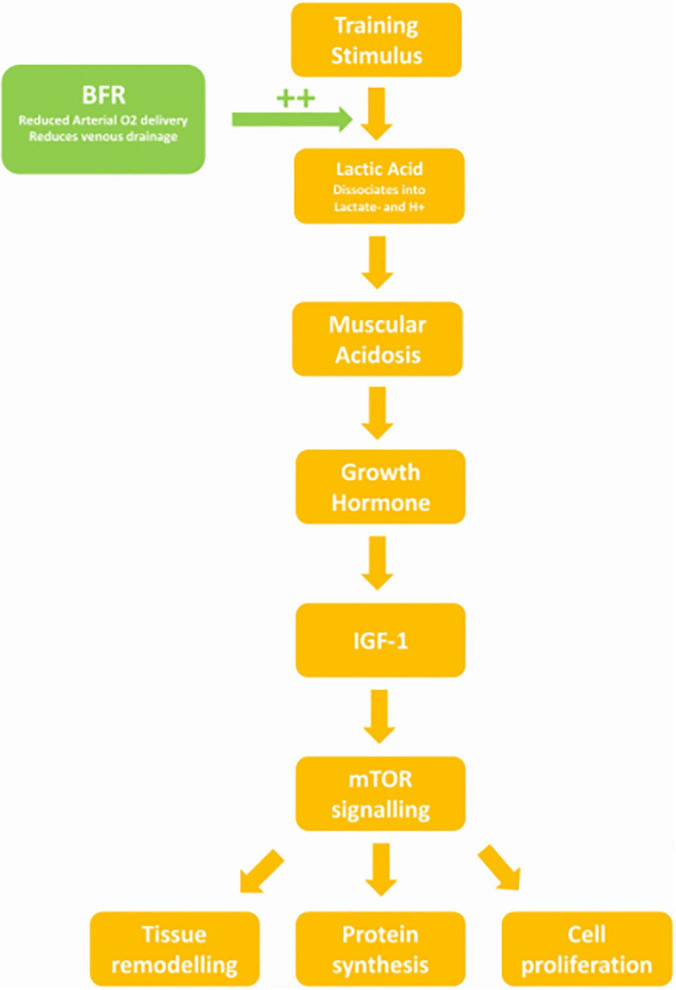
Flowchart demonstrating the physiological effect of BFR.

It is the increased stimulation of GH release by BFR training that likely causes desired strength outcomes. GH when released in pulses (such as after exercise) stimulates IGF-1 release. This increases muscle protein uptake, protein synthesis, and stimulates myoblast and satellite cell proliferation (Florini et al., 1996). Abe’s study found that after even 2 weeks of BFR training at 20%1RM, circulating IGF-1 was 23.8% higher than baseline, whereas the non-BFR matched intensity group saw no significant change (Abe et al., 2005). IGF-1 activates mammalian target of Rapamycin (mTOR), resulting in a mechanism that causes cell division and tissue growth (Feng and Levine, 2010). In Fujita’s study of BFR, subjects training at 20%1RM with a 200 mmHg cuff showed higher levels of lactate and GH than intensity matched non-BFR controls (Fujita et al., 2007). The BFR group had higher levels of Ribosome s6 Kinase phosphorylation (a target of mTOR signaling) and decreased levels of Eukaryotic Translation Elongation Factor 2 phosphorylation (Fujita et al., 2007). This resulted in a 46% increase in protein synthesis (Fujita et al., 2007). As seen above, the %1RM required during BFR to match the metabolic stress of high intensity exercise and elicit this mechanism, appears to be 30%, whereas to exceed high intensity exercise training at 40%1RM or above may be required (Figure 6).

### Blood Flow and Hypoxia

As demonstrated in this review, extremely high BFR cuff pressure (≥100% LOP) resulted in far worse strength outcomes for the BFR group compared to high intensity controls. At the higher occlusion pressures earlier fatigability starts to impair training volume. 180 mmHg of cuff pressure was shown to reduce femoral blood flow by 52% compared to a non-BFR control during exercise (Christiansen et al., 2019). Sundberg et al. reported that higher external cuff pressure reduced blood oxygen delivery to skeletal muscle resulting in lower venous oxygen and higher lactate levels with increasing pressure (Sundberg, 1994). This impairs performance, as maximal voluntary contraction was shown to fall to similar levels between a normoxic training group and a hypoxic group from pre-exercise to exhaustion, however the time for the hypoxic group to reach exhaustion was 56% shorter (Fulco et al., 1996). Exercise studies at low oxygen levels shows significant drops in endurance, dynamic and static strength (Eiken and Tesch, 1984).

### Hypoxia and Fatigue

Blood flow restriction effectiveness may then correlate directly with total volume of work done as this increases GH response and therefore greater strength improvements. Jessee et al. (2018) found that during their eight-week BFR study, subjects training to failure performed far less total volume load at every week at 80% LOP compared to 40% LOP. This might explain why both studies using pressures exceeding total LOP (Cook et al., 2017; de Lemos Muller et al., 2019) found significantly lower strength improvements in leg extension despite their participants training to failure, as early fatigability may have impaired the total repetition volume. Similarly, both studies who both had their BFR group train at high intensity, lower repetition protocols (60–80%1RM, 6–12 reps/set) did not see significantly different results compared to the control (Laurentino et al., 2008; Biazon et al., 2019). Higher loads typically result in greater improvements in 1RM than lower load programs (Schoenfeld et al., 2017). However, their BFR groups trained at much lower rep protocols which may not have been enough volume to produce significant lactic acid levels for GH stimulation. Also reduced muscle oxygenation likely impaired their subject’s ability to train at their actual 80%1RM. Therefore, they did not elicit the metabolic stress required to achieve significant strength gains.

### Confounding Factors

Cuff width between the 21 papers reviewed varied between 3 and 17 cm wide (see Appendix Figure 13). The pressure required to occlude blood flow reduces proportionally with increasing cuff width (Crenshaw et al., 1988; Loenneke et al., 2012). As the cuff width was not standardized between papers, the actual mmHg pressure applied to occlude blood flow would vary appreciably between studies even at the same %LOP. Wider cuffs may also contribute to higher loads being lifted, as the compressive force is spread over a larger area over the muscle. One study looking at compressive gear in powerlifting athletes found significantly higher maximal lifts in bench press, deadlift and squats in athletes using compressive support gear (Michal et al., 2020). It is suggested that the compressive gear such as knee wraps store elastic potential during the eccentric phase of the lift, then returning that energy as mechanical force during the concentric phase of the lift (Harman and Frykman, 1990). High BFR pressures (100–150% LOP) have been shown to significantly increase 1RM strength and repetitions to failure against controls not using external compression (Wilk et al., 2020). A wider cuff would distribute more of this energy across the target muscle, resulting in higher weights being lifted and potentially more strength adaptation stimulation (Michal et al., 2020). Narrow cuffs may cause more localized damage due to higher pressures being focused over a smaller area, as high applied pressures (230 mmHg) during BFR training have been shown to impair hypertrophy at the cuff site (Shaw and Murray, 1982). Other localized damage could be caused to tissues underneath a highly pressurized narrow cuff.

Also, thigh circumference strongly influences LOP and can influence the occlusive effect of a given pressure (Shaw and Murray, 1982). This may be due to the buffering effect of additional tissue and muscle mass between the cuff and vasculature. Composition of the tissue between the cuff and vasculature is also important, as Loenneke demonstrated the different pressure buffering abilities of fat and muscle at different respective tissue thicknesses (Loenneke et al., 2015). Loenneke proposed a formula for calculating arterial occlusion pressure accounting for blood pressure (BP), fat thickness and muscle thickness (Loenneke et al., 2015). This illustrates that body mass, limb CSA and body composition all play a significant role in LOP. It also demonstrates the need for individualized LOP to be applied for BFR training rather than standardized, as actual pressure exerted on the vascular system may vary wildly with cuff width, limb thickness and body composition.

Finally in all the studies discussed, LOP was tested pre training. BP rises significantly during exercise but also with length of session and fatigue (MacDougall et al., 1992). Peak mean BP during exercise has been found to increase with exercise intensity when tested at 50, 70, and 87.5% 1RM (MacDougall et al., 1992). This is attributed to the increased blood flow necessary to complete heavy lifts and the effect of the Valsalva manouevre (MacDougall et al., 1992). Contraction of the core musculature is required to stabilize the spine during heavy lifts. This increases intrathoracic pressure which is transmitted immediately through the arterial tree to the exercising limb. This can cause SBP to far exceed applied LOP, impacting occlusion dose and therefore physiological effect. Future studies into the effect of BFR should attempt to calibrate LOP during rather than pre-exercise, so that the true applied levels of %LOP are recorded.

### Limitations

Studies chosen were not separated by sex of participants, however it has been established in prior literature that there are sex differences in skeletal muscle vasculature (Coutinho et al., 2013), muscle arterial compliance (Coutinho et al., 2013), rate of muscle oxygen desaturation during exercise (Keller and Kennedy, 2021) and degree of muscular and vascular adaptation from a training stimulus (Barnes and Fu, 2018). There is also an observed increase in mean and diastolic BP seen in women during the ovulation and luteal phase of the menstrual cycle (Lutsenko and Kovalenko, 2017) which would affect their minimum LOP during this time, changing their dosage of LOP for a significant period of any intervention lasting over 4 weeks.

Similarly studies were not separated by participant age, however both arterial and venous compliance (Olsen and Länne, 1998) and muscular blood flow during exercise (Lawrenson et al., 2003) reduces significantly with age especially in less active older people. This paper includes studies using participants with and without prior experience of resistance training. There is yet to be studies into whether prior strength training experience may influence the amount of strength gain using BFR with a resistance training program. However, exercise adaptations such as mitochondrial density, muscle capillary density and oxygen uptake (Bassett and Howley, 2000) may also result in differences of physiological effect from BFR training, however this is yet to be studied. All these factors would significantly influence LOP and degree of physiological effect from BFR, as well as training adaptation. Only studies that used a “continuous” method of BFR were used as opposed to intermittent. There is yet to be conclusive proof of a difference in training adaptation and intramuscular physiology between continuous and intermittent BFR (Fitschen et al., 2014; Freitas et al., 2020; Davids et al., 2021), and mixed results on level of perceived exertion and discomfort using either method (Fitschen et al., 2014; Freitas et al., 2019, 2020).

Although we did not control for the above variables, results were consistent between the different demographics and therefore still have validity. However because of this, findings should be qualified by future research due to the heterogeneity of populations. We would have liked to investigate the response to BFR among different patient demographic groups, however the sparseness of literature in this area would now allowed for adequate sampling to make meaningful conclusions.

Due to the strict inclusion criteria the pool of relevant papers became limited (Gavanda et al., 2020). Although several studies into BFR are being conducted in Japan and Brazil, mistranslation of the original articles may have affected results, so were excluded. Papers including specific patient groups could have been considered (i.e., those with cardiovascular/orthopedic/rheumatological conditions). However, factors such as pain and weakness may have confounded results.

This paper reviews the effect of BFR on maximal strength but does not look at its effect on hypertrophy, which may be achieved at different LOP and %1RM combinations than suggested by this review. While most papers studied low intensity training (20–30%1RM) with BFR, few have looked at moderate intensity (40–60%) which is where this review suggests the maximal benefit from BFR may be derived.

## Conclusion

This was the first review that looked at the influence of combinations of pressure dosages (%LOP) and exercise intensity (%1RM) on strength gains during BFR training. Training at a %1RM of 20% or below did not exert enough physiological stress to induce strength improvements. 30%1RM appears to produce results matching non-BFR high intensity training, whereas 40%1RM may produce results exceeding high intensity training. %1RM appears to be a larger contributing factor to strength increases than dosage of LOP. Effect size rose progressively with increasing exercise intensity, whereas intensity matched protocols at moderate and high levels of LOP had no significant difference in strength gain. Significant results for BFR training seem to appear between 50–80% LOP, with the effect dropping off either side of this range due to insufficient metabolic stress or earlier fatigue. More research is needed into the apparent “maximal effect zone” identified in this meta-analysis to get optimal strength improvements from BFR training. Future studies should consider cuff width and thigh circumference when calculating chosen %LOP and should make efforts to test LOP during exercise to ensure adequate vascular occlusion during protocols. Studies should also consider comparing male vs. female participant groups, groups split by age range and resistance training experienced vs. unexperienced groups to see if there is a difference in response between them.

## Data Availability Statement

The original contributions presented in the study are included in the article/Supplementary Material, further inquiries can be directed to the corresponding authors.

## Author Contributions

AD collected the data, interpreted the results, and wrote the manuscript. BP acted as research supervisor and proof reader. Both authors contributed to the article and approved the submitted version.

## Conflict of Interest

The authors declare that the research was conducted in the absence of any commercial or financial relationships that could be construed as a potential conflict of interest.

## Publisher’s Note

All claims expressed in this article are solely those of the authors and do not necessarily represent those of their affiliated organizations, or those of the publisher, the editors and the reviewers. Any product that may be evaluated in this article, or claim that may be made by its manufacturer, is not guaranteed or endorsed by the publisher.
