# Supplementary material for: Is There a Minimum Effective Dose for Vascular Occlusion During Blood Flow Restriction Training?
Source: Front Physiol. 2022 Apr 8;13:838115. doi: 10.3389/fphys.2022.838115 (PMC9024204; doi:10.3389/fphys.2022.838115)
Supplement: Supplementary file 1 [file Data_Sheet_1.docx]

**APPENDIX**

| **Study** | **Level of Evidence**  **^a^** | **Selection Criteria**  **^b^** | **Setting**  **^c^** | **Demographic**  **^d^** | **Limb Composition/Size**  **^e^** | **BFR method^f^** | **Pressure quantified^g^** | **Pressure Rationale**  **^h^** | **Missing Data reporting**  **^i^** | **Total** |
| --- | --- | --- | --- | --- | --- | --- | --- | --- | --- | --- |
| **Bemben et al** | 5 | 1 | 1 | 1 | 0 | 1 | 1 | 1 | 0 | 11 |
| **Biazon et al** | 5 | 1 | 0 | 1 | 0 | 1 | 1 | 0 | 1 | 10 |
| **Brandner et al** | 5 | 1 | 1 | 1 | 1 | 1 | 1 | 1 | 1 | 13 |
| **Centner et al** | 5 | 1 | 1 | 1 | 0 | 1 | 1 | 1 | 0 | 11 |
| **Cook et al** | 5 | 1 | 1 | 1 | 1 | 1 | 1 | 0 | 1 | 12 |
| **De Lemos Muller et al** | 5 | 1 | 1 | 1 | 0 | 1 | 1 | 0 | 1 | 11 |
| **Fahs et al** | 5 | 1 | 1 | 1 | 1 | 1 | 1 | 1 | 1 | 13 |
| **Gavanda et al** | 5 | 1 | 1 | 1 | 1 | 0 | 1 | 1 | 1 | 12 |
| **Hill et al** | 5 | 1 | 1 | 1 | 1 | 1 | 1 | 1 | 1 | 13 |
| **Jessee et al** | 5 | 1 | 1 | 1 | 1 | 1 | 1 | 1 | 1 | 13 |
| **Kim et al** | 5 | 0 | 1 | 1 | 1 | 0 | 1 | 0 | 1 | 10 |
| **Laurentino et al** | 5 | 0 | 1 | 1 | 1 | 1 | 1 | 0 | 0 | 10 |
| **Laurentino et al** | 5 | 0 | 1 | 1 | 1 | 1 | 1 | 0 | 1 | 11 |
| **Letieri et al** | 5 | 1 | 0 | 1 | 1 | 1 | 1 | 0 | 1 | 11 |
| **Lixandrao et al** | 5 | 0 | 1 | 1 | 1 | 1 | 1 | 0 | 1 | 11 |
| **Manimmanakorn et al** | 5 | 0 | 1 | 1 | 1 | 0 | 1 | 0 | 1 | 10 |
| **May et al** | 5 | 1 | 1 | 1 | 1 | 1 | 1 | 1 | 1 | 13 |
| **Mendonca et al** | 5 | 1 | 1 | 1 | 1 | 1 | 1 | 0 | 1 | 12 |
| **Neto et al** | 5 | 1 | 1 | 1 | 1 | 1 | 1 | 1 | 1 | 13 |
| **Rauro et al** | 5 | 0 | 0 | 1 | 0 | 0 | 1 | 0 | 1 | 8 |
| **Vechin et al** | 5 | 1 | 1 | 1 | 1 | 1 | 1 | 0 | 1 | 12 |

**Figure 10. Quality assessment tool used for this review**

1. ***Evidence level calculated using Oxford CEBM score***
2. ***Clear inclusion and exclusion criteria outlined in study***
3. ***Setting clearly described***
4. ***Age, sex and training status of sample group detailed***
5. ***Limb CSA/Circumference/tissue composition measured at start of study***
6. ***Method for determining limb occlusion pressure and study pressure defined***
7. ***%LOP clearly detailed***
8. ***Reasoning for %LOP choice clearly defined***
9. ***Participant dropout or missing data accounted for***

| **Author^a^** | **Year^b^** | ***Title***  ***^c^*** | **Demographic^d^** | **Sample size^e^** | **% LOP**  **^f^** | **%1RM^g^** | **Training Volume**  **^h^** | **Length of study**  **^i^** | **Muscle group**  **^j^** | **1RM change %**  **^k^** | **Control 1RM change %**  **^l^** | **Control protocol^m^** |
| --- | --- | --- | --- | --- | --- | --- | --- | --- | --- | --- | --- | --- |
| **De Lemos Muller et al** | 2019 | *Effects of low-load resistance training with blood flow restriction on the perceived exertion, muscular resistance and endurance in healthy young adults* | Trained young men | 26 | SBP + 20mmHg | 30 | 4 sets x 22 reps 3x/wk | 8 weeks | LL Knee ext | 30.9 +/- 21.6 | **45.7 +/- 22.6*** | HI |
|  |  |  |  |  | SBP - 20mmHg | 30 | 4 sets x 22 reps 3x/wk | 8 weeks | UL Elbow flex | 18.1 +/- 23.2 | 26.9 +/- 23 | HI |
| **Centner et al** | 2019 | *Effects of blood flow restriction training with protein supplementation on muscle mass and strength in older men* | Untrained older adults (>50yo) | 30 | 50 | 20 | 4 sets x 30,15,15,15 2x/week | 8 weeks | LL Knee ext | 10.2 ± 24.8 | - 5.3 ± 8.6 | HI |
| **Rauro et al** | 2019 | *Effects of strength training with and without blood flow restriction on quality of life in elderly women* | Untrained older women (>60yo) | 33 | 70 | 40 | 3 sets x 15 2x/week | 14 weeks | UL Wrist Flex | **55.3 ± 24*** | 49.8 ± 30.3 | HI |
| **Neto et al** | 2019 | *Effects of strength training with continuous or intermittent blood flow restriction on the hypertrophy, muscular strength and endurance of men* | Trained young men | 25 | 80 | 20 | 4 sets x15 2x/week | 6 weeks | UL Bench Press | 0.5 ± 19.8 | -0.1 ± 18.1 | IM |
|  |  |  |  |  | 80 | 20 | 4 sets x15 2x/week | 6 weeks | UL Front Pulldown | 0.3 ± 18.1 | 4.4 ± 23.4 | IM |
|  |  |  |  |  | 80 | 20 | 4 sets x15 2x/week | 6 weeks | UL Elbow ext | 1 ± 19.2 | 0.9 ± 18.9 | IM |
|  |  |  |  |  | 80 | 20 | 4 sets x15 2x/week | 6 weeks | UL Elbow flex | 2 ± 18 | 6.1 ± 19 | IM |
| **Jessee et al** | 2018 | *Muscle adaptations to high-load training and very low-load training with and without blood flow restriction* | Untrained young men | 40 | 40 | 15 | 4 sets to failure 2x/week | 8 weeks | LL Knee ext | 0.3 ± 4.3 | **10.9 ± 4.4*** | HI |
|  |  |  |  |  | 80 | 15 | 4 sets to failure 2x/week | 8 weeks | LL Knee ext | 2.5 ± 4.5 | **10.9 ± 4.4*** | HI |
| **Vechin et al** | 2015 | *Comparisons between low-intensity resistance training with blood flow restriction and high-intensity resistance training on quadriceps muscle mass and strength in elderly* | Untrained older adults (>50yo) | 23 | 50 | 20 | 4 sets x 30,15,15,15 2x/week | 6 weeks | LL Leg Press | 15.8 ± 51.6 | **50.3 ± 58.8*** | HI |
| **Manimmanakorn et al** | 2013 | *Effects of low-load resistance training combined with blood flow restriction or hypoxia on muscle function and performance in netball athletes* | Trained young adults | 30 | 100 | 20 | 3 sets to failure 2x/week | 5 weeks | LL Knee ext | **12.1 ± 7.8*** | 1.0 ± 14.3 | IM |
| **Brandner et al** | 2019 | *Muscular Adaptations to Whole Body Blood Flow Restriction Training and Detraining* | Untrained young adults | 39 | 60 | 20 | 4 sets x 30,15,15,15 3x/week | 8 weeks | LL Knee ext | 20.9 ± 43 | 24.9± 31.3 | HI |
|  |  |  |  |  | 60 | 20 | 4 sets x 30,15,15,15 3x/week | " | LL Squat | 10.5 ± 27 | **16.2 ± 25.9*** | HI |
|  |  |  |  |  | 60 | 20 | 4 sets x 30,15,15,15 3x/week |  | LL Calf Raise | 8.6 ± 29 | 17.8 ± 31 | HI |
|  |  |  |  |  | 60 | 20 | 4 sets x 30,15,15,15 3x/week |  | UL Bench Press | 5.7 ± 25.8 | 13.2 ± 36.8 | HI |
|  |  |  |  |  | 60 | 20 | 4 sets x 30,15,15,15 3x/week |  | UL Seated Row | 5.5 ± 31 | **15.4 ± 27.3*** | HI |
|  |  |  |  |  | 60 | 20 | 4 sets x 30,15,15,15 3x/week |  | UL Elbow flex | 11.3 ± 35.2 | 13.3 ± 26.6 | HI |
| **Cook et al** | 2017 | *Blood Flow Restricted Resistance Training in Older Adults at Risk of Mobility Limitations* | Untrained older adults (>50yo) | 36 | 150 | 30 | 3 sets to failure 2x/week | 12 weeks | LL knee ext | 24.7 | **57.6*** | HI |
|  |  |  |  |  | 150 | 30 | 3 sets to failure 2x/week |  | LL knee flex | 19.9 | 24.4 | HI |
|  |  |  |  |  | 150 | 50 | 3 sets to failure 2x/week |  | LL Leg Press | 15.9 | 19.2 | HI |
| **Gavanda et al** | 2020 | *Low-intensity blood flow restriction calf muscle training leads to similar functional and structural adaptations than conventional low-load strength training: A randomized controlled trial* | Trained young men | 21 | 60 | 30 | 4 sets to failure 2x/week | 6 weeks | LL Calf Raise | 24.5 ± 25.8 | 20.9 ± 20.9 | HI |
| **Biazon et al** | 2019 | *The Association Between Muscle Deoxygenation and Muscle Hypertrophy to Blood Flow Restricted Training Performed at High and Low Loads* | Untrained young men | 30 | 100 | 20 | 3 sets x20 2x/week | 10 weeks | LL Knee ext | 18.7 ± 49.9 | 36.9 ± 23.4 | HI |
|  |  |  |  |  | 100 | 80 | 3 sets x10 2x/week | 10 weeks | LL Knee Ext | 33.8 ± 39 | 36.9 ± 23.4 | HI |
| **Bemben et al** | 2019 | *Can Blood Flow Restricted Exercise Improve Ham:Quad Ratios Better Than Traditional Training* | Trained young women | 14 | 50 | 30 | 4 sets x 30,15,15,15 3x/week | 6 weeks | UL Bench Press | 16.4 ± 11.4 | 24.4 ± 4.3 | HI |
|  |  |  |  |  | 50 | 30 | 4 sets x 30,15,15,15 3x/week | 6 weeks | UL Front Pulldown | 23.8 ± 16.2 | 28.1 ± 11.9 | HI |
|  |  |  |  |  | 50 | 30 | 4 sets x 30,15,15,15 3x/week | 6 weeks | UL elbow flex | 37.2 ± 11.4 | 29.3 ± 19.1 | HI |
|  |  |  |  |  | 50 | 30 | 4 sets x 30,15,15,15 3x/week | 6 weeks | LL Leg Press | 19.0 ± 5.9 | 18.2 ± 8.7 | HI |
|  |  |  |  |  | 50 | 30 | 4 sets x 30,15,15,15 3x/week | 6 weeks | LL Knee ext | 27.2 ± 12.5 | 17.0 ± 6.3 | HI |
|  |  |  |  |  | 50 | 30 | 4 sets x 30,15,15,15 3x/week | 6 weeks | LL Knee flex | 27.5 ± 11.6 | 23.7 ± 8.8 | HI |
| **Fahs et al** | 2014 | *Muscular adaptations to fatiguing exercise with and without blood flow restriction* | Untrained middle age | 18 | 50 | 30 | 3 sets to failure 2x/week | 6 weeks | LL Knee ext | 19.7 ± 30.1 | 16.4 ± 30.2 | IM |
| **Letieri et al** | 2018 | *Effect of 16 weeks of resistance exercise and detraining comparing two methods of blood flow restriction in muscle strength of healthy older women: A randomized controlled trial* | Untrained older women (>60yo) | 56 | 80 | 30 | 4 sets x 15 3x/week | 16 weeks | LL Knee ext | 27.8 ± 15.4 | 26.5 ± 15.8 | HI |
|  |  |  |  |  | 80 | 30 | 4 sets x 15 3x/week | 16 weeks | LL Knee flex | 36.7 ± 21.2 | 35 ± 17.8 | HI |
|  |  |  |  |  | 45 | 30 | 4 sets x 15 3x/week | 16 weeks | LL Knee ext | 15.7 ± 14.9 | 26.5 ± 15.8 | HI |
|  |  |  |  |  | 45 | 30 | 4 sets x 15 3x/week | 16 weeks | LL Knee flex | 22.8 ± 20.3 | 35 ± 17.8 | HI |
| **Laurentino et al** | 2012 | *Strength Training with Blood Flow Restriction Diminishes Myostatin Gene Expression* | Untrained young men | 29 | 80 | 20 | 3 sets x 15 2x/week | 8 weeks | LL Knee ext | 40.10 | 36.20 | HI |
| **May et al** | 2018 | *Lower body blood flow restriction training may induce remote muscle strength adaptations in an active unrestricted arm* | Untrained young men | 24 | 60 | 50 | 3 sets x 10 3x/week | 7 weeks | UL Elbow flex | **16.9*** | 4.3 | IM |
|  |  |  |  |  | 60 | 30 | 4 sets x 30,15,15,15 3x/week | 7 weeks | LL Knee ext | **23*** | 10.3 | IM |
|  |  |  |  |  | 60 | 30 | 4 sets x 30,15,15,15 3x/week | 7 weeks | LL Knee flex | **16.8** | 14.6 | IM |
| **Lixandrao et al** | 2015 | *Effects of exercise intensity and occlusion pressure after 12 weeks of resistance training with blood-flow restriction* | Untrained young men | 26 | 40 | 20 | 3 sets x 15 2x/week | 12 weeks | LL Knee ext | 10.3 | **21.6*** | HI |
|  |  |  |  |  | 80 | 20 | 3 sets x 15 2x/week | 12 weeks | LL Knee ext | 13.2 | **21.6*** | HI |
|  |  |  |  |  | 40 | 40 | 3 sets x 15 2x/week | 12 weeks | LL Knee ext | 12.2 | **21.6*** | HI |
|  |  |  |  |  | 40 | 80 | 3 sets x 15 2x/week | 12 weeks | LL Knee ext | 12.7 | **21.6*** | HI |
| **Laurentino et al** | 2007 | *Effects of Strength Training and Vascular Occlusion* | Untrained young men | 16 | 100 | 80 | 3 sets x 6 2x/week | 8 weeks | LL knee ext | 34.5 ± 21.8 | 36.9 ± 23.8 | HI |
|  |  |  |  |  | 100 | 60 | 3 sets x 12 2x/week | 8 weeks | LL Knee ext | 35.3 ± 17..7 | 37.6 ± 18.3 | HI |
| **Hill et al** | 2019 | *Low-load blood flow restriction elicits greater concentric strength than non-blood flow restriction resistance training but similar isometric strength and muscle size* | Untrained young women | 30 | 40 | 30 | 4 sets x 30,15,15,15 3x/week | 4 weeks | UL Elbow flex | **36.9*** | 25.8 | IM |
| **Mendonca et al** | 2021 | *Contralateral training effects of low-intensity blood-flow restricted and high-intensity unilateral resistance training* | Untrained young adults | 30 | 60 | 20 | 4 sets x 30,15,15,15 5x/week | 4 weeks | LL Calf Raise | 16 | 17 | HI |
| **Kim et al** | 2017 | *Low-load resistance training with low relative pressure produces muscular changes similar to high-load resistance training* | Untrained young men | 14 | 50 | 30 | 4 sets x 30,15,15,15 3x/week | 8 weeks | UL elbow flex | 20.3 | 24.5 | HI |

**Figure 11. Key characteristics of included studies for this review. * denotes statistical difference between BFR and control group 1RM change (*p<0.05). NB Although De Lemos Muller et al did not use a clear %LOP, there was individualisation of occlusion pressure, so this study was included.***

1. ***First author of study***
2. ***Year of publication***
3. ***Title of article***
4. ***Age group, sex and resistance training experience of participants***
5. ***Total sample size***
6. ***%LOP applied with cuff for BFR group***
7. ***%1RM intensity trained at by BFR group during intervention***
8. ***Sets, repetitions and frequency of training sessions for BFR group***
9. ***Length of exercise intervention***
10. ***Muscle group trained during protocol***
11. ***%1RM change in BFR group with standard deviation where given***
12. ***%1RM change in control group with standard deviation where given***
13. ***Control group protocol. HI = High intensity 70-80%1RM moderate repetition. IM= Intensity and volume matched to BFR group***

| **Author** | **% AOP** | **%1RM used** | **Effect BFR** | **Exercise** | **Reason for missing effect size data** |
| --- | --- | --- | --- | --- | --- |
| **Bemben et al** | 50 | 30 | 0.723499 | chest press |  |
| **Bemben et al** | 50 | 30 | 1.181424 | lat pull |  |
| **Bemben et al** | 50 | 30 | 1.535715 | bicep curl |  |
| **Bemben et al** | 50 | 30 | 1.541407 | leg press |  |
| **Bemben et al** | 50 | 30 | 1.447646 | Leg ext |  |
| **Bemben et al** | 50 | 30 | 1.350694 |  |  |
| **Biazon et al** | 100 | 80 | 1.053418 |  |  |
| **Biazon et al** | 100 | 20 | 0.473522 |  |  |
| **Brandner et al** | 60 | 20 | 0.560776 | leg ext |  |
| **Brandner et al** | 60 | 20 | 0.388826 | squat |  |
| **Brandner et al** | 60 | 20 | 0.3181 | calf raise |  |
| **Brandner et al** | 60 | 20 | 0.197334 | chest press |  |
| **Brandner et al** | 60 | 20 | 0.172723 | seated row |  |
| **Brandner et al** | 60 | 20 | 0.326818 | bicep curl |  |
| **Centner et al** | 50 | 20 | - |  | 1 |
| **Cook et al** | 150 | 30 | - | leg ext | 1,2 |
| **Cook et al** | 150 | 30 | - | leg curl |  |
| **Cook et al** | 150 | 50 | - | leg press |  |
| **De Lemos Muller et al** | SBP + 20mmHg | 30 | 1.311868 | leg ext |  |
| **De Lemos Muller et al** | SBP - 20mmHg | 30 | 0.785511 | bicep curl |  |
| **Fahs et al** | 50 | 30 | 0.625661 |  |  |
| **Gavanda et al** | 60 | 30 | 1.010636 |  |  |
| **Hill et al** | 40 | 30 | 0.921 |  |  |
| **Jessee et al** | 40 | 15 | 0.076923 |  |  |
| **Jessee et al** | 80 | 15 | 0.538462 |  |  |
| **Kim et al** | 50 | 30 |  |  | 2 |
| **Laurentino et al** | 100 | 60 | 1.726579 |  |  |
| **Laurentino et al** | 80 | 20 |  |  | 2 |
| **Laurentino et al** | 100 | 80 | 1.606695 |  |  |
| **Letieri et al** | 80 | 30 | 1.623825 | leg ext |  |
| **Letieri et al** | 80 | 30 | 1.926033 | leg flex |  |
| **Letieri et al** | 45 | 30 | 1.023298 | leg ext |  |
| **Letieri et al** | 45 | 30 | 1.296235 | leg flex |  |
| **Lixandrao et al** | 40 | 20 | 0.420071 |  |  |
| **Lixandrao et al** | 40 | 40 | 0.526946 |  |  |
| **Lixandrao et al** | 40 | 80 | 0.5664 |  |  |
| **Lixandrao et al** | 80 | 20 | 0.687324 |  |  |
| **Manimmanakorn et al** | 100 | 20 |  |  | 1 |
| **May et al** | 60 | 50 | 2.627942 | bicep curl |  |
| **May et al** | 60 | 30 |  |  | 1 |
| **May et al** | 60 | 30 |  |  | 1 |
| **Mendonca et al** | 60 | 20 | 1.416354 |  |  |
| **Neto et al** | 80 | 20 | 0.024503 | chest press |  |
| **Neto et al** | 80 | 20 | 0.015683 | lat pull |  |
| **Neto et al** | 80 | 20 | 0.046784 | tricep ext |  |
| **Neto et al** | 80 | 20 | 0.115667 | bicep curl |  |
| **Rauro et al** | 70 | 40 | 2.776072 |  |  |
| **Vechin et al** | 50 | 20 | 0.33538 |  |  |

***F*igure 12. Effect sizes calculated for all 48 protocols using Cohen’s d. Exercise used in intervention included where multiple muscle groups were tested in the same study. Reason for missing data 1. Pre and/or post intervention 1RM not included in study. Reason for missing data 2. Standard deviations for 1RM result not included in study.**

| **Author** | **Year** | **Title** | **Length of study (weeks)** | **Session/week** | **Reps per session** | **Total reps over study** | **Cuff Width (cm)** | **BFR BM (kg)** | **Control BM (kg)** |
| --- | --- | --- | --- | --- | --- | --- | --- | --- | --- |
| **De Lemos Muller et al** | 2019 | Effects of low-load resistance training with blood flow restriction on the perceived exertion, muscular resistance and endurance in healthy young adults | 8 | 3 | 88 | 2112 | 17 | 77 | 79.37 |
| **Centner et al** | 2019 | Effects of blood flow restriction training with protein supplementation on muscle mass and strength in older men | 8 | 2 | 75 | 1200 | 12 | 84.9 | 80.6 |
| **Rauro et al** | 2019 | Effects of strength training with and without blood flow restriction on quality of life in elderly women | 14 | 2 | 45 | 1260 | N/A | 68.78 | 69.69 |
| **Neto et al** | 2019 | Effects of strength training with continuous or intermittent blood flow restriction on the hypertrophy, muscular strength and endurance of men | 6 | 2 | 60 | 720 | 6 | 67.5 | 78 |
| **Jessee et al** | 2018 | Muscle adaptations to high-load training and very low-load training with and without blood flow restriction | 8 | 2 | 4F | - | 10 | 68.4 | - |
| **Vechin et al** | 2015 | Comparisons between low-intensity resistance training with blood flow restriction and high-intensity resistance training on quadriceps muscle mass and strength in elderly | 6 | 2 | 75 | 900 | 18 | 72.55 | - |
| **Manimmanakorn et al** | 2013 | Effects of low-load resistance training combined with blood flow restriction or hypoxia on muscle function and performance in netball athletes | 5 | 2 | 74 | 740 | 5 | 65.2 | - |
| **Brandner et al** | 2019 | Muscular Adaptations to Whole Body Blood Flow Restriction Training and Detraining | 8 | 3 | 75 | 1800 | 10.5 | 72.5 | 71.1 |
| **Cook et al** | 2017 | Blood Flow Restricted Resistance Training in Older Adults at Risk of Mobility Limitations | 12 | 2 | 3F | - | 6 | 74.3 | - |
| **Gavanda et al** | 2020 | Low-intensity blood flow restriction calf muscle training leads to similar functional and structural adaptations than conventional low-load strength training: A randomized controlled trial | 6 | 2 | 52 | 624 | 7 | 82.6 | 83.9 |
| **Biazon et al** | 2019 | The Association Between Muscle Deoxygenation and Muscle Hypertrophy to Blood Flow Restricted Training Performed at High and Low Loads | 10 | 2 | 60 | 1200 | 17.5 | 72.7 | - |
|  |  | | 10 | 2 | 30 | 600 | 17.5 | 72.7 | - |
| **Bemben et al** | 2019 | Can Blood Flow Restricted Exercise Improve Ham:Quad Ratios Better Than Traditional Training | 6 | 3 | 75 | 1350 | 13.5 | 66 | 59 |
| **Fahs et al** | 2014 | Muscular adaptations to fatiguing exercise with and without blood flow restriction | 6 | 2 | 3F | - | 5 | 82.7 | - |
| **Letieri et al** | 2018 | Effect of 16 weeks of resistance exercise and detraining comparing two methods of blood flow restriction in muscle strength of healthy older women: A randomized controlled trial | 16 | 3 | 60 | 2880 | - | 68 | 69.5 |
| **Laurentino et al** | 2012 | Strength Training with Blood Flow Restriction Diminishes Myostatin Gene Expression | 8 | 2 | 45 | 720 | 17.5 | 72.1 | 73.8 |
| **May et al** | 2018 | Lower body blood flow restriction training may induce remote muscle strength adaptations in an active unrestricted arm | 7 | 3 | 30 | 630 | 10.5 | 73 | 72.4 |
|  |  | | 7 | 3 | 75 | 1575 | 10.5 | 73 | 72.4 |
| **Lixandrao et al** | 2015 | Effects of exercise intensity and occlusion pressure after 12 weeks of resistance training with blood-flow restriction | 12 | 2 | 45 | 1080 | 17.5 | 77.4 | 74.9 |
| **Laurentino et al** | 2007 | Effects of Strength Training and Vascular Occlusion | 8 | 2 | 18 | 288 | 14 | 80.15 | 71.4 |
|  |  | | 8 | 2 | 36 | 576 | 14 | 80.15 | 71.4 |
| **Hill et al** | 2019 | Low-load blood flow restriction elicits greater concentric strength than non-blood flow restriction resistance training but similar isometric strength and muscle size | 4 | 3 | 75 | 900 | 3 | 60.1 | 60.6 |
| **Mendonca et al** | 2021 | Contralateral training effects of low-intensity blood-flow restricted and high-intensity unilateral resistance training | 4 | 5 | 75 | 1500 | 13 | 64.7 | 61.9 |
| **Kim et al** | 2017 | Low-load resistance training with low relative pressure produces muscular changes similar to high-load resistance training | 8 | 3 | 75 | 1800 | 5 | 75.9 | 73.7 |

**Figure 13. Confounding factors of studies reviewed, including study length, exercise prescription, cuff width, and body mass (BM) of BFR and control group.**
